# Mental illness in Bwindi, Uganda: Understanding stakeholder perceptions of benefits and barriers to developing a community-based mental health programme

**DOI:** 10.4102/phcfm.v9i1.1462

**Published:** 2017-11-30

**Authors:** Kristen L. Sessions, Lydia Wheeler, Arya Shah, Deenah Farrell, Edwin Agaba, Yusufu Kuule, Stephen P. Merry

**Affiliations:** 1 Mayo Clinic School of Medicine, Rochester, United States; 2 Bwindi Community Hospital, Kanungu, Uganda; 3 Department of Family Medicine, Mayo Clinic, Rochester, United States

## Abstract

**Background:**

Mental illness has been increasingly recognised as a source of morbidity in low- and middle-income countries and significant treatment gaps exist worldwide. Studies have demonstrated the effectiveness of task sharing through community-based treatment models for addressing international mental health issues.

**Aim:**

This paper aims to evaluate the perceptions of a wide range of mental health stakeholders in a Ugandan community regarding the benefits and barriers to developing a community-based mental health programme.

**Setting:**

Bwindi Community Hospital (BCH) in south-west Uganda provides services through a team of community health workers to people in the Kanungu District.

**Methods:**

Thematic analysis of 13 semi-structured interviews and 6 focus group discussions involving 54 community members and 13 mental health stakeholders within the BCH catchment area.

**Results:**

Stakeholders perceived benefits to a community-based compared to a hospital-based programme, including improved patient care, lower costs to patients and improved community understanding of mental illness. They also cited barriers including cost, insufficient workforce and a lack of community readiness.

**Conclusions:**

Stakeholders express interest in developing community-based mental health programmes, as they feel that it will address mental health needs in the community and improve community awareness of mental illness. However, they also report that cost is a significant barrier to programme development that will have to be addressed prior to being able to successfully establish such programming. Additionally, many community members expressed unique sociocultural beliefs regarding the nature of mental illness and those suffering from a psychiatric disease.

## Introduction

Studies on the effects of deinstitutionalisation of mental health services have revealed both success and failures with regard to the treatment of mental illness.^1^ However, task sharing models, involving the provision of resources to expand the health workforce through widespread training and dissemination of non-physician healthcare workers, are being increasingly studied for their potential to address the global shortage of mental health services through the development of community-based mental health programmes.^2,3,4^ Though mental illness is being increasingly recognised as a source of morbidity in low- and middle-income countries, significant treatment gaps continue to exist throughout the world, limiting access to mental health services for most persons.^5^

Programmes such as Emerging Mental Health Systems in Low-and Middle-Income Countries (EMERALD) in Ethiopia, India, Nepal, Nigeria, South Africa and Uganda and the Programme for Improving Mental Health Care (PRIME) in Ethiopia, India, Nepal, South Africa and Uganda seek not only to address the human resource barrier by training community health workers (CHWs) in mental healthcare delivery but also to overcome cultural barriers by tailoring programmes to the specific needs and beliefs of a community.^6,7^ Other efforts to increase mental health services include integration of care into primary care delivered by general providers and decentralisation of services.^8,9,10^

However, in working to develop effective community-based mental health programming, challenges, including deeply rooted stigmas and a lack of knowledge about mental illness, serve as critical barriers to implementation.^8,11,12^ Studies have stressed the importance of understanding country-specific sociocultural nuances that affect perceptions and treatment of mental health prior to developing country-specific programming. Stigma, poverty and lack of familiarity with mental illness among healthcare providers have been shown to decrease efficacy of treatment.^13,14,15,16,17,18^ In contrast, interventions developed within communities and tailored to community contexts have increased both efficacy of and satisfaction with the programme.^4,7,12,19^

In 2013, the World Health Organization published the WHO Mental Health Action Plan, which outlines four key objects with regard to combating the global mental health crisis. These objectives include (1) strengthening leadership for mental health, (2) providing comprehensive, integrated and responsive services in the community, (3) implementing health promotion and disease prevention mental health services and (4) augmenting information systems and research on mental health. The goal of this comprehensive action plan was to protect fundamental human rights and to empower communities. In the present study, we partner with the Bwindi Community Hospital (BCH) in Uganda, which aims to implement the WHO objectives through its creation of a community mental health programme.^20,21^

Though mental health has been reported to be a national priority in Uganda’s Ministry of Health’s strategic plan, only half of the patients with mental illness seek care.^22,23^ Limited access to care has been attributed to scarce funding, concentration of services within urban areas and stigmatisation of people with mental illness.^24,25^ One study reported only 1.13 human resources in mental health per 100 000 individuals and only 28 psychiatrists in all of Uganda.^8^ Over 60% of available psychiatric beds are located in or near Kampala, despite 87.7% of the population living in rural areas.^22,26^

The high concentration of hospitals and psychiatric providers in major cities in Uganda raises the question of how to provide mental health resources to individuals in rural communities who comprise the majority of Uganda’s population. For this reason, community-based treatment models may better address the mental health needs in Uganda by bringing resources directly into communities.

In a previous study, we outlined perceptions of community members with regard to mental illness in general. However, in the process of developing community-based mental health programming, it is important to also understand the perceptions of mental health stakeholders that will be vital in implementing and providing such services. In this paper, we explore perceptions of a wide range of mental health stakeholders in a Ugandan community regarding the benefits and barriers to developing a community-based mental health programme. This paper is the second in a series that ultimately aims to examine factors important to development of a community mental health programme using the Bwindi Community Mental health programme as an example.^27^

## Methods

### Setting and study design

Uganda is a country of 37.5 million people in East Africa. It is estimated that up to one-third of the population suffers from some form of mental illness.^22,28^ BCH is a 112-bed hospital in south-west Uganda that provides medical care and community health services to a catchment population of 252 100 people in the Kanungu District.^29^ BCH is staffed by 121 personnel onsite, including doctors, nurses, midwives and support staff. The BCH community programme includes 502 village health workers, who visit communities daily as needed.

The majority of people served by BCH live on less than $1/day in a farming village and are of Bakiga ethnicity; a minority are indigenous Batwa peoples, who were displaced from the Bwindi Impenetrable Forest in 1991 when the government designated the forest a national park.^29,30^

The mental health services programme is integrated into the community health programme as well as the hospital and outpatient clinic. Established in 2013, the goal of the programme is to provide outreach and rehabilitation services to 3150 members of the community living with mental illness and their families by the end of 2020.^31^ The programme includes a community psychology graduate (PhD) who heads the team, a psychiatric clinical office assistant, enrolled mental health nurse (assessment, treatment and community follow-ups), public health office assistant (review of patients), three community health nurses (administering of medications in the hospital) and access to the 502-person volunteer village health worker team (help with identification, referral and follow-up of community individuals). Together with generalist physicians and nurses, these outpatient care providers provide care for individuals suffering from psychosis, affective disorders, substance abuse and epilepsy. In addition, hospital administrators from human resources, hospital services, communications and clinical services contribute to the programme development through fundraising, staffing and programme design. The clinical and administrative staff members interviewed are referred to in the rest of this paper as ‘hospital stakeholders’ (Tables 1 and 2).

**TABLE 1:** Demographics of community participants.

Community	Participants	Male or female
Buhoma	6 lay community members 1 community leader 1 service user or caregiver	4 males 4 females
Kanyamasinga	5 lay community members 1 community leader 1 service user or caregiver 1 community health worker	4 males 4 females
Kanyashande	5 lay community members 2 community leaders 4 service users or caregivers 1 community health worker	5 males 7 females
Kyumbugusho	8 lay community members 1 service user or caregiver	4 males 5 females
Mukono	5 lay community members 1 community leader 2 service users or caregivers 1 community health worker	4 males 5 females
Nkwenda	2 lay community members 2 community leaders 2 service users or caregivers 2 community health workers	4 males 4 females

**TABLE 2:** Demographics of hospital administrative participants.

Administrative stakeholders	Description of role	Male or female
Head of Hospital Services	Responsible for functioning of entire hospital and community outreach programme.	1 male
Head of Human Resources	Responsible for hiring additional mental health staff	1 male
Communications Administrators (2)	Responsible for fundraising and communications to hospital partners and local communities.	1 male 1 female
Psychologist	Author of the Community Mental Health proposal, head of current mental health programme.	1 male
Mental Health Nurse	Conducts mental health patient visits in both a hospital and community setting.	1 male
Head of Clinical Services	Oversees all clinical services at the hospital. Direct patient care in sexual and reproductive health.	1 male
International Physician	Oversees the general inpatient hospital service and performs informal mental health screenings.	1 female
Local Physician	Works in outpatient and inpatient medicine including acute care for mentally ill.	1 male
Community Health Worker Administrator	Train CHWs and oversee the development of community education programmes.	1 male
Community Health Workers (3)	Responsible for following patients in the community, screening for disease and medication delivery.	3 males

CHW, community health worker.

Participants for community focus groups were identified in two ways. We sought to achieve census-level representation across adults and communities. Six communities were purposively chosen to allow for this: Buhoma, Kanyamasinga, Kanyashande, Kyumbugusho, Mukono and Nkwenda. This allowed for theme saturation and contextual variation as verified through the data analysis process. For each community, seven households were randomly selected from cross-sectional census surveys (BCH 2010; BCH 2013). The purpose of the study was discussed with the adults of the household and consent to participate in the focus group was sought. Thirty-three of 36 adults (91.7%) consented. Secondly, participants including those who declined were asked to recommend other individuals who may not have been initially identified by the research team but may be instrumental in helping communities decide if and how to treat mentally ill persons. Twenty-one of 25 (84%) adults recommended consented. Focus group discussions (FGDs) were conducted with community members, referred to as ‘community stakeholders’ below, in villages within the BCH catchment area during the same time period by author E.A. (Table 1). CHWs or ‘village health teams’ also helped to identify, refer to the clinic or hospital and follow up individual patients in the community. Although they function in this fashion as part of the BCH healthcare team, their views on mental illness as noted below appear to parallel those of their community at large so their responses are included with the ‘community stakeholders’.

These hospital and community stakeholders were then interviewed in a series of semi-structured interviews (SSIs) and FGDs using a SSI we developed in collaboration with our Ugandan co-investigators for use at the local level. All SSIs were conducted in English. We were unable to conduct focus groups with non-community members because of the temporal challenges of coordinating comparable gatherings of administrators, healthcare providers and hospital stakeholders within our study time frame. Although the use of SSIs and FGDs hinders the ability to fully compare and contrast the data from the two groups, SSIs have been shown to be an effective way of gaining qualitative information about perceptions of individuals.^32^ Because of the significantly skewed ratio of non-community member stakeholders to community member stakeholders, multiple SSIs were preferred over a single FGD. Theme saturation was verified through the data analysis process. All FGDs were conducted in Rukiga, the local language, and then translated to English. English is the national language of Uganda and spoken in the hospital by staff. However, many community members speak limited English. For this reason, SSIs with staff members were conducted in English but community FGDs were conducted in Rukiga.

### Data collection

In total, 13 SSIs and 6 FGDs (each consisting of 8–12 participants) were conducted. SSIs were chosen because they have been shown to be an effective way of gaining qualitative information about perceptions of individuals.^32^

The 6 FGDs involving 54 community stakeholders (i.e. community leaders, mental health service users, caregivers, lay community members) were conducted in 6 different communities. The only individuals present at these gatherings were the participants of the FGD and at least three of the authors.

### Data analysis

Interview data were de-identified and transcribed verbatim in Rukiga. This was back-translated to English by three Rukiga–English interpreters to ensure that contextualised meaning was preserved. Data were managed with Nvivo 10 software and content was analysed based on techniques outlined by Tesch and Maykut and Morehouse.^33,34^ All data were kept de-identified and stored on an encrypted hard drive. All transcripts were read once initially to provide an overview of the tone and scope of the information to the authors. The transcripts were coded using inductive thematic analysis and a framework analysis approach.^32,35^ In the first round of analysis, a minimum of three authors independently came up with thematic labels for each transcript. The team then came together to compare and revise codes in an iterative fashion. Overarching themes were further refined and subcoded. Using this approach, the investigators collectively agreed upon themes and sub-themes within the texts in relation to the objectives of the study. The coded material was then checked by English–Rukiga interpreters for veracity and consistency.

### Ethical consideration

Our project was coordinated with BCH staff, who wished to gather information on community and health sector perceptions for planning the subsequent steps in initiating such a project. BCH has its own quality improvement protocol and our research team was provided approval through this entity. Our study was deemed institutional review board (IRB) exempt by the Mayo Clinic Institutional Review Board and the leadership and staff of BCH based on the standard language utilised by IRBs worldwide, including the Makerere and Mbarara official Ugandan IRBs and Uganda National Council for Science and Technology (UNCST)-accredited Research Ethics Committees. Written informed consent was obtained from all the participants.

## Results

The participants interviewed in the 13 SSIs included 13 BCH employee mental healthcare stakeholders (Table 1). Emergent themes identified from responses of BCH workers and community stakeholders are outlined and compared below.

### Hospital stakeholders

Across all the SSIs, the hospital staff was supportive of a community-based mental health programme. The hospital staff identified many potential benefits to a community-based compared to a hospital-based programme including improved patient care, increased sustainability, lower costs to patients and improved community understanding of mental illness.

Most of the hospital staff, both clinical and administrative, identified improved patient care as the most important reason to develop a community-based mental health programme. They asserted that treating patients in their homes and observing their home environment would increase understanding of the patient’s condition and unique needs, improving both accuracy of diagnosis and their treatment.

‘The best environment to recognize behavior is in their niche, not at a facility. Someone will come in, they are bathed. … They will put on their clothes.… But you want to see in the environment, at home, how does this person sleep? What does he eat? Is there a way for him to take his medication? So we want to get to the root cause of the problem because we know that most of mental health problems are psychosocial. There is an interaction between the mind and environment. And we cannot examine that interaction without seeing it.’ (Interviewee 7, male, Head of Clinical Services)

‘The patient is being managed in their environment where they are familiar with and that increases the healing process.’ (Interviewee 1, male, Head of Hospital Services)

‘If someone fails to come back for review, [*the team*] will follow you up, even the village health team or community nurses will know so we will not easily lose patients.’ (Interviewee 3, female, Communications Administrator)

Hospital staff acknowledged that increasing community participation in the treatment of mental illness would help to provide community education on the topic of mental illness and to reduce stigma associated with its diagnosis and treatment.

‘If the eventual goal of care is to enable patients with mental illness to be fully functional, contributing members of society, acceptance by their community is essential for healing … we return to the community and say, the people you are writing off can actually do something.’ (Interviewee 1, male, Head of Clinical Services)

‘[*Community care*] helps reduce stigma by de-mystifying mental health patients and not reinforcing the concept that all mental health patients should be locked away in asylums. Part of managing mental health problems is enabling patients to function within society, and this is easier to achieve if patients are able to stay within their community.’ (Interviewee 8, female, International Physician)

Hospital staff also frequently recognised challenges facing community mental health programming, including insufficient workforce and a lack of community awareness of mental illness.

‘We need to ensure that we have staff that will be able to … go out into the community and we need to have people to be on standby when the people are in the community because there may be cases here that need attention.’ (Interviewee 4, male, Communications Administrator)

‘Once you enroll in the mental programme and you go back into the community they will look at you as someone who is unfit and you will be segregated from the community.’ (Interviewee 9, male, Local Physician)

Bwindi Community Hospital is a primarily donor-funded hospital, receiving significant support from non-profits and donors in the United States, England and other developed countries. Several administrators expressed concerns over the cost of a new programme because of variability of funding from month to month.

‘Mental health treatment is very expensive. The people we serve here are very poor. They can hardly afford the cost of treatment so at times they are unable to complete their visits because they are very expensive and drugs are very expensive. So basically, the program, for it to continue, it needs funding. It needs support. And that support is not there as of now.’ (Interviewee 3, female, Communications Administrator)

‘There are very few [*donors*] who want to identify themselves with that need. It’s not really attractive.… [*Support*] does not come as easily as it does looking for support for children or women.’ (Interviewee 3, female, Communications Administrator)

‘We [*the hospital*] are taking on some kind of cost, but it is not comparable to this person, what they would spend, if you add up the total sum, including missing a day of work. If it is a child, the mother has to miss a day of work to bring the child, plus the transport, on the way they will take a bottle of soda and a donut. All that is cost. So that, in order to minimise the waste of monies for these people who are not even income earning, we should send care into the community.’ (Interviewee 7, male, Head of Clinical Services)

### Community stakeholders

In comparison to hospital staff, many community stakeholders expressed concerns regarding the idea of individuals with mental health issues being treated in the community. They also frequently expressed their belief that the hospital in Bwindi was the appropriate place for those with mental illness to receive treatment before returning to their communities.

Community members frequently expressed their belief that the hospital was the only appropriate place for the treatment of mental illness, as it separates those who are mentally ill from the rest of the community while they are being treated.

‘Once we have established that a person has mental illness, he/she should be taken to hospital, stay there and only be brought back home once they have been completely cured and fully treated.’ (Nkwneda participant 3, male, community member)

‘They should ideally have a place where they are confined and not just wandering everywhere.’ (Nkwenda participant 6, female, community member)

‘BCH should take persons to the hospital; confine them from there until they get back to their senses.’ (Mukono participant 1, male, community leader)

Several community stakeholders cited specific examples of individuals they knew who had benefited from the mental health services provided at BCH.

‘We had a mad person in Mukono who was mad for a long time, was also half dressed, recently went to the hospital of Bwindi and is now doing well. She can now dig, wash clothes, and other activities. It’s from this that we go convinced that these diseases can be treated.’ (Nkwenda participant 5, female, community member)

‘There are some we have seen who have gotten services and have come back to normal.’ (Buhoma participant 1, female, community member)

While the community groups mainly favoured hospital-based care, individuals did acknowledge that a community-based mental health programme would train and empower the CHWs to provide additional services for the families for whom they already provide care. They acknowledged that a community programme would be beneficial with regard to facilitating continuity of care through more easily providing medications, and follow-up.

‘If the health providers can know him in our community, you can try to follow up such that it can help us.’ (Buhoma participant 3, female, community member)

‘We have a program that reaches every village that is connected up to a household level so no one is missed.’ (Interviewee 10, male, CHW Administrator)

‘It increases the chances that the person will actually finish their treatment.’ (Interviewee 10, male, CHW Administrator)

Finally, community stakeholders frequently expressed their beliefs that mental illness was the result of witchcraft, Satan’s work or punishment for wrongdoings and therefore should properly be managed by traditional and faith healers. In contrast, the medical professionals interviewed were hesitant to blame spiritual forces for illness that may have a biological basis and therefore benefit and be well controlled by aggressive medical treatment. Community members also expressed their belief that persons with mental illness should be isolated from society.

‘A mentally ill person does not have rights because his mind is not okay. She/he has no rights.’ (Kanyamasinga participant 1, female, community member)

‘Sometimes when children go to school to study, when a student become very bright, his/her friends can bewitch him/her causing mental illness.’ (Kanyamasinga participant 1, female, community member)

‘…Others whose parents/relatives invite satanic powers/demons to come and disorganize them, which ends up making their children (relatives) get mental illness.’ (Nkwneda participant 4, female, community member)

‘The reason why you cannot befriend a mad person is that their acts are beshaming. So, if you found that you have befriended a mad person, you are also mad.’ (Nkwneda participant 8, female, community member)

## Discussion

Hospital stakeholders interviewed in this study expressed strong support for a community-based mental health programme. The participants identified significant potential for improvement of care of patients and eventual increased acceptance of mental illness within the community. Their perceptions of benefits of a community-based programme and their belief in the importance of decreasing stigma are in line with previous literature on the subject.^3,9,10,13^ However, they also cited associated cost as being a major barrier to the development of such programming. Potential solutions include marketing community-based care to prospective donors (e.g. using success stories of patients whose lives have been transformed through community-based care) and continuing to pursue government support (e.g. partnering with the national referral hospital and national initiatives that provide community-based services).

Community stakeholders all advocated for a hospital-based programme where patients are isolated from other patients. They demonstrated that significant stigma about mental illness remains in the community which fosters fear and discrimination. This fear and misunderstanding leads to significant social isolation of patients with mental illness. While a community-based programme may eventually help reduce stigma allowing patients to be accepted within their communities, some groundwork is needed before such a programme will be accepted. Education is needed to garner the support of the community before implementing a community-based programme. Proposed efforts for community education include radio programmes, community meetings and increased education of the community and CHWs by mental health clinic staff. Education efforts should focus on the various types of mental illness and their causes, their signs and symptoms and living examples of how treatment of patients with mental illness can enable them to lead normal and productive lives in society. By demystifying mental illness, fear in the community will decrease and social inclusion will increase.

Community perceptions of mental illness include a predominant perception that such problems have a spiritual aetiology and therefore will most benefit from a traditional or faith healer intervention. Education in conjunction with traditional healers and local clergy may be most effective in designing a successful collaborative community-based programme.

## Limitations

One limitation of the study is that individuals with mental illness and their family members were not interviewed as an individual focus group. While three of the focus groups had individuals who self-identified as either having a mental illness or having a family member with mental illness, these groups were not necessarily proportionately represented. We cannot rule out the influence of group dynamics on the individuals’ contribution to the discussion. Groups should be considered as key stakeholders and given a voice in future programme development. Future research could focus on addressing the perceptions, needs and wishes of those directly affected by mental illness. However, their limited representation in this study does not diminish the opinions of those whose opinion we sought – the hospital and CHW staff who must design and implement the community-based programme and the community – their community – who must accept them back into their society, supporting them in their healing, restoring their dignity and giving them hope.

## Conclusion

Stakeholders at BCH and in the Bwindi community recognise the need for improved care for mental illness. There is strong support for a community-based mental health programme throughout the hospital staff. This effort is strongly supported by the literature as an appropriate and effective model for mental healthcare. While interest is strong, cost is still a significant barrier to programme development and additional support is needed, which likely must come from government and international donors. There is also a clear need for community education about mental illness to debunk prevalent myths and to reduce stigma, enabling reintegration of those being treated for mental illness into their communities.
